# Research and Evaluation on an Optical Automatic Detection System for the Defects of the Manufactured Paper Cups

**DOI:** 10.3390/s23031452

**Published:** 2023-01-28

**Authors:** Ping Wang, Yang-Han Lee, Hsien-Wei Tseng, Cheng-Fu Yang

**Affiliations:** 1 College of Artificial Intelligence, Yango University, Fuzhou 350015, China; 2 Department of Electrical and Computer Engineering, Tamkang University, New Taipei City 251, Taiwan; 3 Department of Chemical and Materials Engineering, National University of Kaohsiung, Kaohsiung 811, Taiwan; 4 Department of Aeronautical Engineering, Chaoyang University of Technology, Taichung 413, Taiwan

**Keywords:** paper cup, optical automatic detection system, non-contact detection, processing technique

## Abstract

In this paper, the paper cups were used as the research objects, and the machine vision detection technology was combined with different image processing techniques to investigate a non-contact optical automatic detection system to identify the defects of the manufactured paper cups. The combined ring light was used as the light source, an infrared (IR) LED matrix panel was used to provide the IR light to constantly highlight the outer edges of the detected objects, and a multi-grid pixel array was used as the image sensor. The image processing techniques, including the Gaussian filter, Sobel operator, Binarization process, and connected component, were used to enhance the inspection and recognition of the defects existing in the produced paper cups. There were three different detection processes for paper cups, which were divided into internal, external, and bottom image acquisition processes. The present study demonstrated that all the detection processes could clearly detect the surface defect features of the manufactured paper cups, such as dirt, burrs, holes, and uneven thickness. Our study also revealed that the average time for the investigated Automatic Optical Detection to detect the defects on the paper cups was only 0.3 s.

## 1. Introduction

Prior research showed that the people in Taiwan consumed at least 2 billion paper cups in one year, and the global population consumed as many as 250 billion paper cups in one year [1]. The traditional factories of the paper products can only identify the defects through visual detection, and the average manual detection time is about 2 to 3 s. However, the detection qualities are inconsistent among different inspectors, and the detection error rate is as high as 10% because of visual fatigue, which results in the unstable qualities of the manufactured paper products. Recently, with the development of Industry 4.0, only the uses of automated productions are unable to meet the requirements of the modern production processes, while the technologies of machine vision have played a very important role in modern industrial automatic productions for the detection of the products’ surface qualities [2,3]. Therefore, the Automatic Optical Detection (AOD) technologies of the machine visions have been boomed to process the detections of the defects on the manufactured products [4,5].

Image processing technologies can be applied in many fields, and most importantly, they can be used to enhance product quality detection. Image processing technologies are generally divided into three stages: low-level processing, middle-level processing, and high-level processing [6]. The low-level processing can increase the contrast, sharpen the images, and reduce the image noise, and the middle-level processing focuses on image segmentation, object description, and object classification. The high-level processing can center on understanding a group of recognized objects and on performing the vision-related cognition functions, and cognition is defined as the understanding of human-like artificial intelligence [7]. The Gaussian Filter is a filter for signal processing [8], and it is realized by convolving the 2D Gaussian distribution function with the processed images [9]. Gaussian Filter is a kind of low-pass filter and it can remove the high-frequency noise interferences and smooth the images and it has the properties of generating no overshoot to a step function input while the rise and fall time is minimized.

The Sobel operator is an important technology to be used in processing images and computer visions, particularly when it is combined with edge detection algorithms; it can put emphasis on an image’s edges. Therefore, it is used to strengthen the edges of the detection images for the enhanced recognition of the boundary edges between two regions of pixels with different grayscale values [10]. When the grayscale value contrast is larger, the edges’ contours or line features become clearer. The Sobel operator is a discrete differential operator, and when it is combined with the Gaussian smoothing and differential derivation operations, the obtained result is the gradient approximation. There are two different masks for Sobel edge detection: the horizontal mask and the vertical mask, and the Sobel operator can convolve an entire photo in both horizontal and vertical directions. The original image is convolved with a 3 × 3 horizontal mask (*Magnitude_x_*) and is also convolved with a 3 × 3 vertical mask (*Magnitude_y_*). Furthermore, the *Magnitude_x_* and *Magnitude_y_* can be defined as:

(1) $${\mathrm{Magnitude}}_{x}=\left[f\left(x+1,y-1\right)+2f\left(x+1,y\right)+f\left(x+1,y+1\right)\right]-\left[f\left(x-1,y-1\right)+2f\left(x-1,y\right)+f\left(x-1,y+1\right)\right]$$

(2) $${\mathrm{Magnitude}}_{y}=\left[f\left(x-1,y-1\right)+2f\left(x,y-1\right)+f\left(x+1,y-1\right)\right]-\left[f\left(x-1,y+1\right)+2f\left(x,y+1\right)+f\left(x+1,y+1\right)\right]$$

When the Pythagorean Equation (3) is applied, the overall edge of the pixel can be calculated using the horizontal and vertical gradient approximation:

(3) $${\mathrm{Magnitude}}_{edge}=\sqrt{{\mathrm{Magnitude}}_{x}^{2}+{\mathrm{Magnitude}}_{y}^{2}}$$

and the edge angle can be calculated using Equation (4):

(4) $${\mathrm{Angle}}_{edge}=atan2\left({\mathrm{Magnitude}}_{x},{\mathrm{Magnitude}}_{y}\right)$$

Binarization, also called gray-level binary differentiation or threshold value, is the technology applied to distinguish the difference between the target and the background in an image and is often used as image segmentation [11] or is applied to convert a grayscale image into a black-and-white image [12]. In other words, there are only two brightness values (gray levels): white (which can be defined as 255) and black (which is 0) for binarization. If the grayscale critical value is set to *T*, as long as the grayscale value is smaller than *T*, it is all black; otherwise, it is all white. In this way, the target and the background in an image will have significantly different grayscales. For example, if the image is supposed to be f(*x*, *y*), a suitable grayscale critical value *T* is selected in the grayscale range (0 to 255), then f_t_(*x*, *y*) is defined as the segmented image, as shown in Equation (5):

(5) $$f_{t}\left(x,y\right)=\{\begin{array}{c} \;\;0\;\;\;\mathrm{if}\;\;f\left(x,y\right)\leq T\\ 255\;\;\;if\;\;f\left(x,y\right)>T \end{array}$$

After the binarization process, the similarity of the gray value between the target pixel and the background pixel represents its connectivity. There are two commonly used types of connected components, namely the 4-connected component and the 8-connected component [13]. The method of the connected component can quantify and extract the region properties in an image, and then objects in the image can be automatically detected, labeled, and measured. This method of connected components can also group the pixels based on their connectivity to improve the shortcomings of blob detection methods; therefore, it is also used in this study. One can judge whether the pixels are connected through the pixels between neighboring relationships. The 4-connected or 8-connected methods are used as the judgment criterion, and eventually, the adjacent pixels can be divided into the same block.

In the manufacturing factories and industries of paper cups, most of the defects of the moulding paper containers are detected by manual visual detection. However, the consistency of detection cannot be precisely controlled due to frequent fatigues caused by the overuse of visions. The purpose of this paper was to investigate an AOD system to detect the defects of the produced paper cups, because this technology can maintain the stability of detection quality, overcome fatigue associated with long work hours, and effectively increase the detection rate. The important innovation of this research mainly focused on the combination of a self-assembly imaging capture system and four pieces of image processing software. The investigated system architecture included an industrial camera, a ring light source, an infrared backlight, a computer, and machine vision software. The image acquisition methods used in this study were divided into three categories: internal image acquisition, external image acquisition, and bottom image acquisition. The Sobel operator can be used in image processing, particularly because it creates an image that emphasises edges within edge detection algorithms [14,15]. In digital signal processing, a Gaussian filter has the property of having no overshoot to a step function input, which can minimize the rise and fall time [16,17]. A connected component (labeling) can be used in computer vision to detect connected regions in binary digital images, although color images and data with higher dimensionality can also be processed [18,19]. However, there are almost no papers that combine these three algorithms to investigate the technologies for detecting defects in paper products. Another important innovation is that the Sobel operator, Gaussian filter, and connected component were used to enhance the recognition effects of the images captured by our self-assembled detection system. We would show that, through the analysis of the image processing results of the investigated system, the defects of the paper cups could be detected automatically. Also, the paper would show that the dirt, holes, uneven thickness, and burrs in disposable paper cups were really detected in the investigated system. The paper would also show that, for the investigated technology, the detection rate of the defects in the paper cups could reach 100%. Compared with the detection time of about 2.5 s for manual detection, there is a far greater difference of 8.3 times. Finally, the investigated system has been tested to determine its timeliness and accuracy in the laboratory and on the production line, respectively. The system actually runs on the detection of paper cups and detects tens of thousands of them on the production line, which has a detection accuracy rate of 99.7%.

## 2. Research Methods

### 2.1. System Architecture

**Figure 1:**
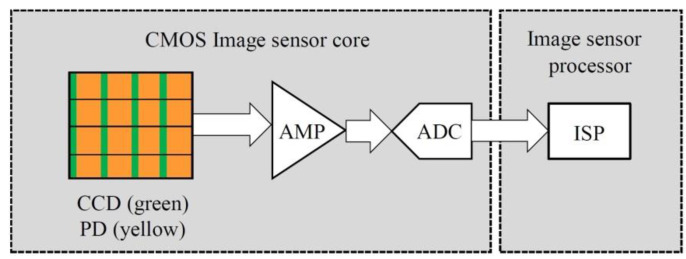
CMOS image sensor architecture.

To capture the images is the first step to recognizing the defects existing in the manufactured paper cups. An image sensor used in this study was composed of a multi-grid pixel array, and each pixel contained a CMOS (complementary metal-oxide semiconductor) and a PD (photo detector) [20]. CMOS image sensors always integrate analog front end (AFE) circuits because this can achieve the properties of reduced cost, fast response, and low power consumption [21]. As shown in Figure 1, when the shutter was activated, the photodetector received the light signal and converted it into an electrical signal. Furthermore, the analog signal was amplified and converted to a digital format for the images’ signal processing. A combined ring light source is composed of three colors of LED light sources, including red (R), green (G), and blue (B), at different angles. When the RGB colors are added according to different proportions, the generated light will produce various colors [22], and as all the RGB color lights are adjusted to their maximum values, the generated light appears white. When the light source is used to highlight the details of the objects’ surfaces, the addition of a reflective light guide plate can reduce the reflective effects of the tested objects. Infrared (IR) is an invisible light, and its wavelength is between visible light and microwave, and it can penetrate measuredly thinner objects [23]. The IR LED matrix panel provides IR light evenly distributed in the backlight, which can highlight the outer edges of the objects for detection with constant strong light. The combined ring light source and IR light were integrated in the investigated system to provide the required detection light source.

**Figure 2:**
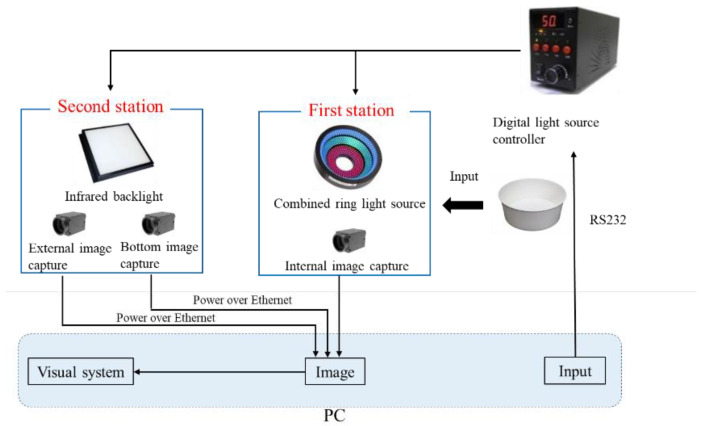
Schematic diagram of the investigated architecture.

The experiment used a machine vision system, and the whole architecture is shown in Figure 2. Two detection stations were employed in this system, one was to detect the internal images by using the combined ring light resource, and the other was to detect the external and bottom images by using the IR backlight. A digital light source controller was used to adjust the brightness of the combined ring light source and IR backlight, which were connected to the computer through the RS232 communication interface, and then the computer could send an order to the light source controller. The PoE (Power over Ethernet) was used to connect with the computer to capture the images from the camera, and then the captured images were processed by the Gaussian filter, Sobel operator, Binarization process, and connected component to recognize the defects of the produced paper cups.

### 2.2. Methods to Capture the Images

**Figure 3:**
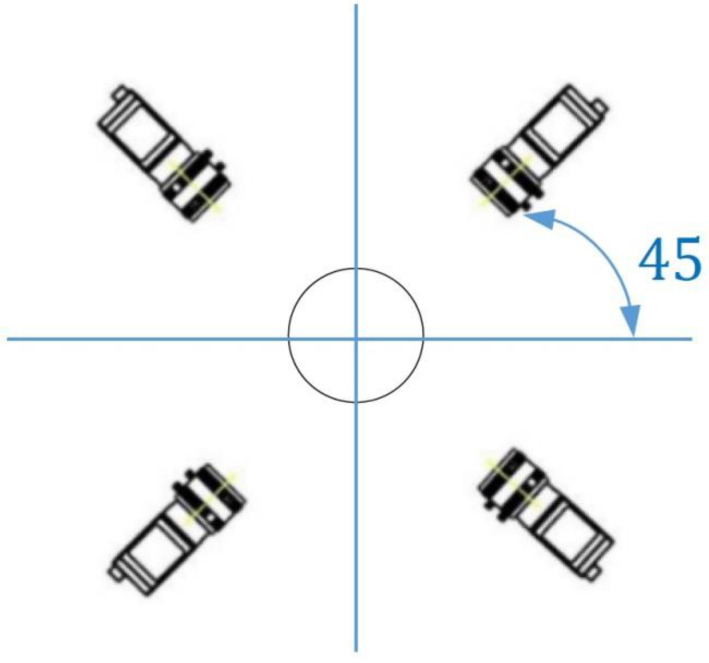
Schematic diagram of 1/4 environment for the camera.

**Figure 4:**
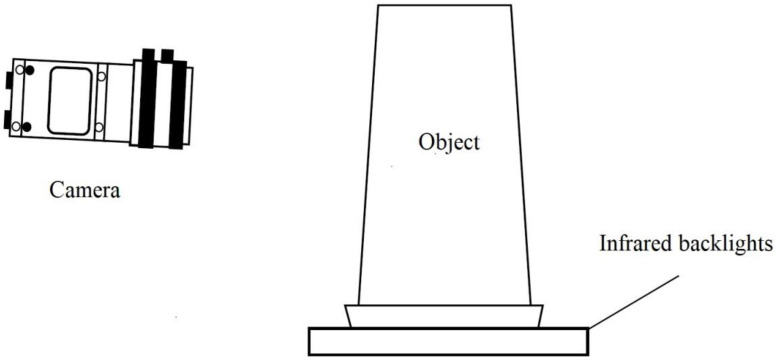
Schematic diagram of the external image capturing environment.

**Figure 5:**
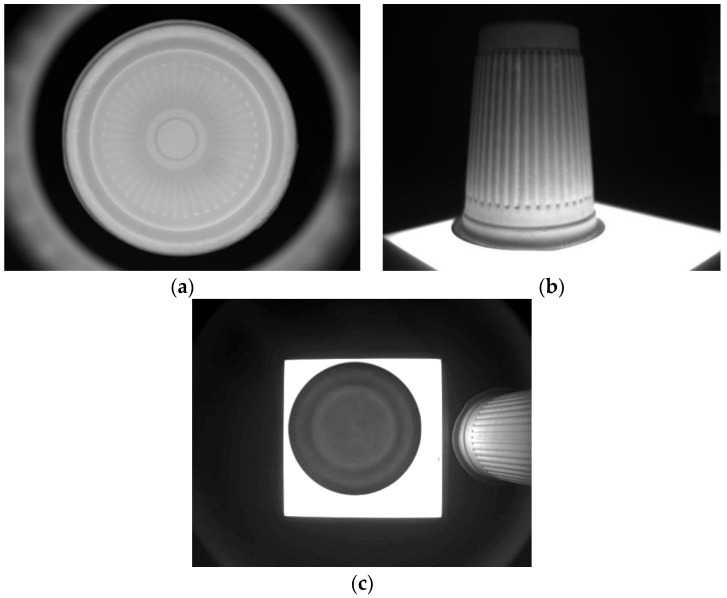
Images of (**a**) internal capturing, (**b**) external capturing, and (**c**) bottom capturing of a detected paper cup.

This research used disposable paper cups as the targets, and the investigated system could capture three different images, including internal, external, and bottom images. In order to capture the internal images, the camera was used to take inside shots of the objects directly for further analysis. The combined ring light source was used as the assistance light source of the camera to capture the inside images of the detected objects. Four cameras were used for the captures of the external images, as shown in Figure 3, and each camera captured a quarter of the outside images of the detected objects. For the reason that the captured four images had similar results, they were classified into one category. There was an IR backlight under the measured object for the enhancements of the captures of the external images, as shown in Figure 4, and the capturing processes of the bottom images were taken in the same environment as the capture processes of the external images. After the lens was facing down to shoot at the bottom, the cameras had taken the images of the detected paper cups in six directions, and therefore, the paper cups were taken with 3D movement. The images for the internal capturing, external capturing, and bottom capturing of a paper cup are shown in Figure 5a, Figure 5b, and Figure 5c, respectively.

## 3. Image Processing Results and Discussion

### 3.1. Measurement of the Defect Areas

**Figure 6:**
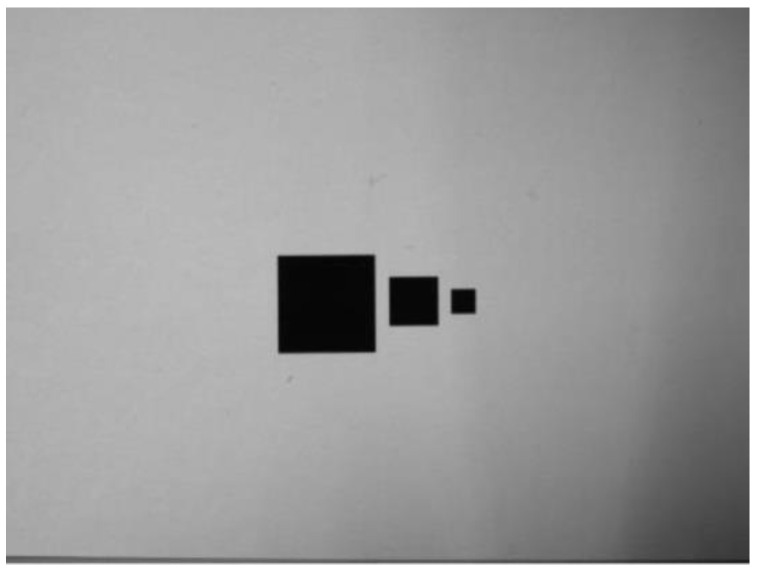
Shadows with 25 mm^2^, 100 mm^2^, and 400 mm^2^ to simulate the defect targets.

**Figure 7:**
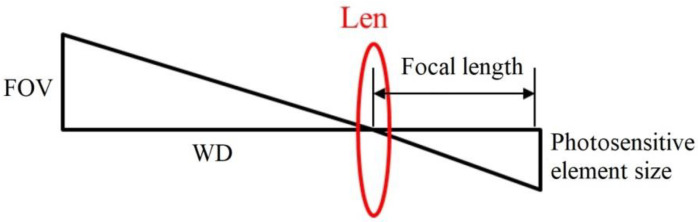
Schematic diagram for using the optical lens to identify the defect area.

This paragraph is mainly to prove the image capture effects of the different defect areas by using the investigated technology, so the shadows of different areas are used to simulate the defects and the investigated technology is used to measure the image areas. In this condition, the frontal light was used as the source, the distances from the lens to the objects (shadows) were in the range of 310–400 mm, and shadows with 25 mm^2^, 100 mm^2^, and 400 mm^2^ were used to simulate the defect targets, as shown in Figure 6. In order to confirm the detection ability of this self-developed system, it is necessary to use shadows of different sizes to identify the ability. Therefore, in Figure 6, three shadows of different sizes are used as detection targets. As shown in Figure 7, FOV (Field of View) was the size of the actual range of the camera’s captured image, which was calculated by three parameters: the WD (working distance), SD (the size of the photosensitive element, width or height), and FL (the focal length of the lens). FOV can be calculated by using Equation (6):

(6) $$\mathrm{FOV}=\frac{\mathrm{WD}\times \mathrm{SD}}{\mathrm{FL}}$$

The resolution (horizontal or vertical) measurement method is defined in Equation (7):

(7) $$\mathrm{Rol}=\frac{\mathrm{FOV}}{\mathrm{IPV}}$$

where IPV is the image width (or length) pixel value. Under the frontal light condition, the effective pixel is one, and the AP value (accuracy pixel) measurement method is defined in Equation (8):

(8) AP=Rol×1

When the defect pixel was identified, the DA (defect areas, mm^2^) were calculated by Equation (9), where the DP value is the number of defective pixels and the PPAA is the pixel accuracy area (mm^2^/pixels). The size of the CMOS sensor in this experiment was 1/1.8 inch (width: 7.2 mm × height: 5.4 mm), the lens focal length was 16 mm, and the camera’s pixels were 1600 × 1200.

(9) DA=DP×PAA

The measurement results of the defect areas showed that under the frontal light environment, when the WD values of 310 to 400 mm were the actual calculation range, the inspection rates of the shadows with 25 mm^2^, 100 mm^2^, and 400 mm^2^ were 100%. As Table 1, Table 2 and Table 3 show, all the pixel areas (pixels) decreased as the WD value increased. For the actual shadow areas of 25 mm^2^, 100 mm^2^, and 400 mm^2^, the identified defect areas were in the ranges of 22.3–22.7 mm^2^, 22.3–22.7 mm^2^, and 22.3–22.7 mm^2^, and the average errors of the defect areas were 9.8%, 8.2%, and 7.8%, respectively. However, all the identified areas are smaller than those of the actual shadow areas, and the average errors of the defect areas decreased as the area of the simulated shadows increased.

### 3.2. Image Processing Method

**Figure 8:**
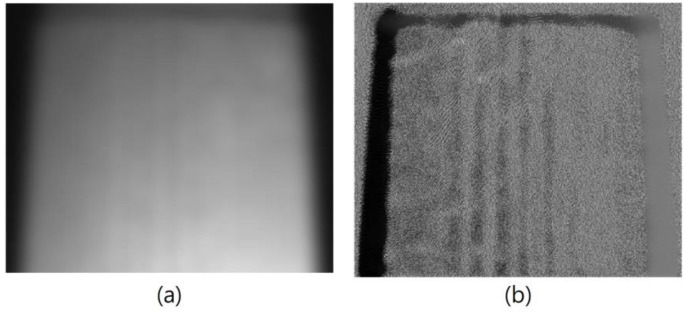
(**a**) Original image and (**b**) Sobel’s *Angle_edge_* result of the paper cups.

The template matching technique is a high-level machine vision technique used in digital image processing to identify the small parts on an image that match a predefined template [24,25]. The template matching technique is expected to address the issue of providing a reference image of a template image and the detected input image we want to recognize. It means that a template is used to detect whether it and the target have the same lines, curves, directions, etc. The Sobel operator applies two variables to create an image with emphasising edges: *Magnitude_edge_* and *Angle_edge_*, the former is used in most cases, and the *Angle_edge_* is used to process the outside images of the paper cups. Figure 8a shows the original image of a paper cup, which shows that the image on the surface of a paper cup was unclear. In order to filter out the misjudgment caused by the vertical texture on the object to be tested, Figure 8a uses a Gaussian filter to expand the horizontal pixels to a large extent, which can reduce the influence of stripes on the recognition results. Figure 8b shows the image of a paper cup after the Sobel’s *Angle_edge_* process, in which the paper cup had an obviously uneven surface. These results prove that Sobel’s *Angle_edge_* process can make the uneven surface smoother and improve the uneven brightness problems, which can enhance the image recognition effect.

**Figure 9:**
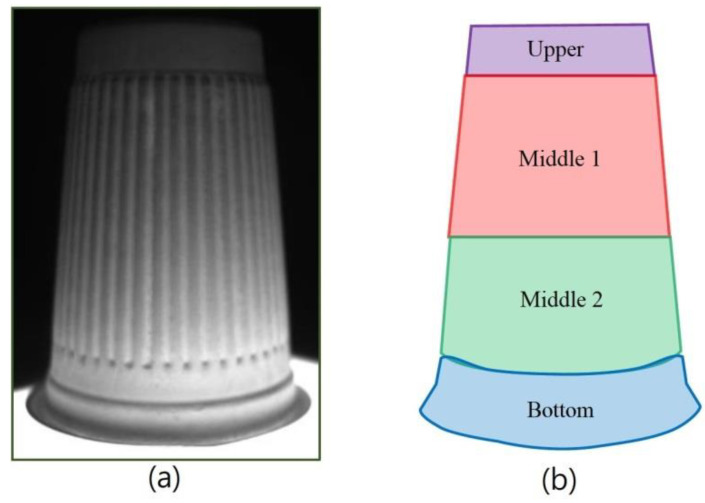
(**a**) Original external image of the cup and (**b**) schematic diagram of image segmentation.

**Figure 10:**
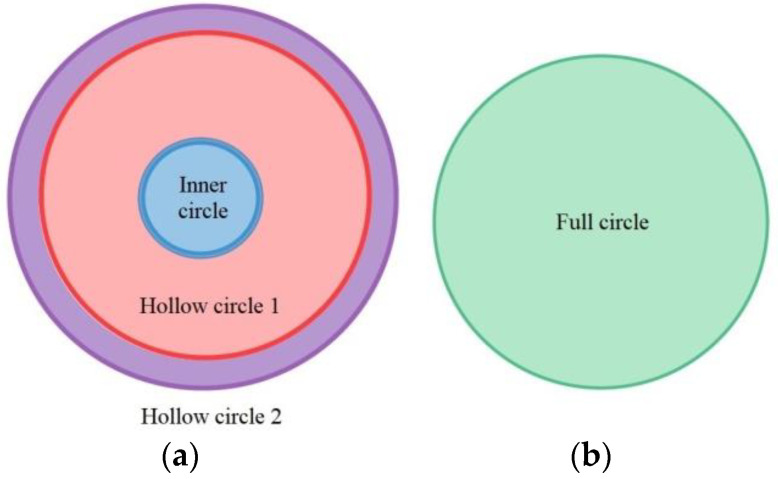
Schematic diagram of image segmentation of a cup (**a**) inner circle, hollow circle 1, and hollow circle 2, (**b**) full circle.

There are two major problems with this investigated detection technology. The first is mainly for interior and bottom detections. When the captured images are processed using the Sobel filter, the edges of all objects will become more obvious. For example, when an image is processed by the Sobel filter, both the regions of the defects and edge outlines will present a white color. In order to prevent misjudgments of the recognition defects during the processing steps, the edge contour must be separated. The second problem is that, from the perspective of external detections, the surface of the object to be tested is closer to the IR backlight; as the surface lowers, the brightness increases. However, this problem can be well improved by using the image’s segmentation processes, which also need to be further processed by Gaussian filtering. As shown in Figure 9, the internal image of the bowl was segmented into pieces. As shown in Figure 10, the inner circle and hollow circle 1 of the image segmented inside the cup were used to detect dirt, the hollow circle 2 was used to detect burrs, the full circle was used to detect holes, and both the outer segmentation image and bottom segmentation image were mainly used to detect the uneven thickness.

**Figure 11:**
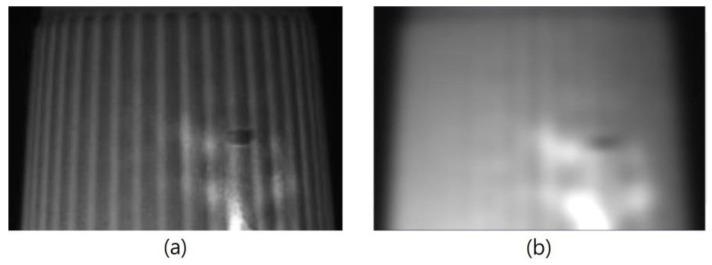
(**a**) Original image and (**b**) Gaussian filter processing result of the outer middle area of a paper cup.

**Figure 12:**
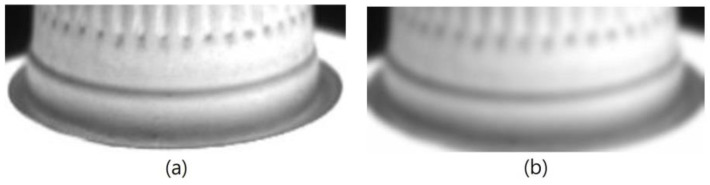
(**a**) Original image and (**b**) Gaussian filter processing result of the outer bottom area of a paper cup.

Manufactured paper cups have the problem of obvious uneven surfaces; however, the uneven surface will have different shadows, which may cause the identification errors. Therefore, the uneven brightness problem needs to be improved for further identification of the defects in the manufactured paper cups. In this study, Gaussian filtering was used to make the uneven surfaces of the captured images smoother, and as the results show in Figure 11, the blemishes did not disappear due to the smoothness process. Therefore, the process of Gaussian filtering does not obscure the defects; it does not increase the chance of identification errors. Gaussian filtering can also be used to improve the uneven brightness problem on a paper cup. For example, there is a problem of uneven brightness at the bottom area when the external image of the paper cup was segmented, as Figure 12a shows. Through the Gaussian filtering, as Figure 12b shows, the bright, uneven surface was effectively diffused.

**Figure 13:**
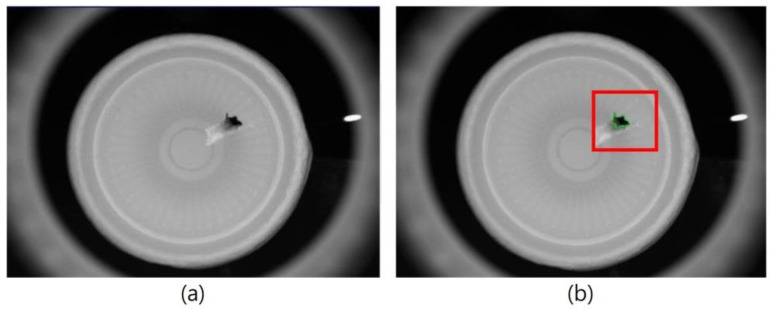
(**a**) Original image and (**b**) the binarization image of the hole inside a paper cup, the red box is used to mark the hole.

In order to separate the defects and the background in the captured images, the binarization process was used to convert the grayscale image to a binary image. The detection pictures in this study came from mass-produced products in a factory, and the environment in which the pictures were taken was uniform and simple. This study used 50 image samples as a benchmark, and the critical value was defined by manual adjustment to achieve a balance between yield and output. When the gray value is smaller than the defined critical value, the region is defined as background and displayed as black; when the gray value is larger than the defined critical value, the region is displayed as white, which is used to display dirt, burrs, and uneven thickness. As shown in Figure 13, if the gray value is smaller than the defined critical value, it is displayed as black and is the background; if the gray value is larger than the defined critical value, it is displayed as white and is a hole. This process allows the blemishes in the image to have a significantly greater grayscale difference as compared with the background.

### 3.3. Image Processing Procedure

**Figure 14:**
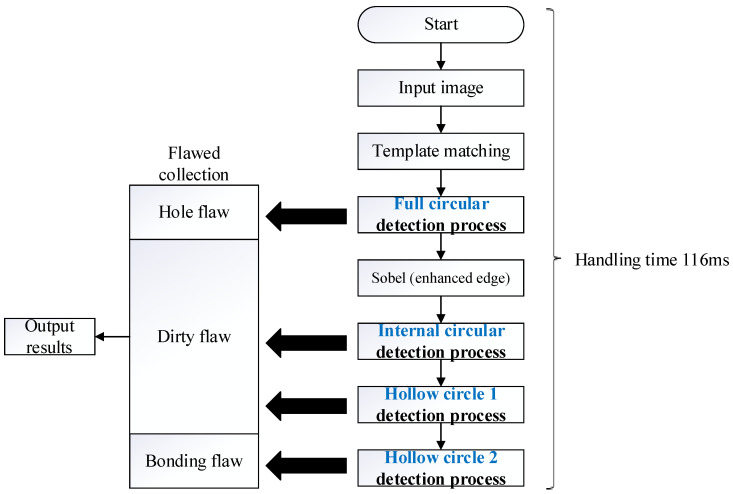
Internal detection processes of the paper cups.

**Figure 15:**
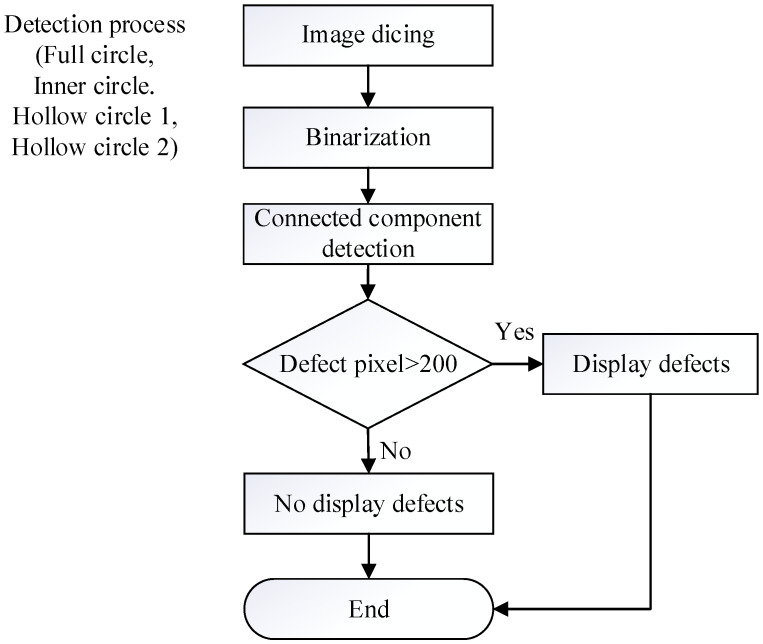
Detailed image processing for each internal detection step.

The internal detection processes of the manufactured paper cups are shown in Figure 14. There were three detection procedures for the manufactured paper cups, which are divided into internal, external, and bottom image detection processes, and each has two processes. First, the captured image template is matched with the target, and the search paper cup target is mostly locked. Furthermore, the full-circle detection process is carried out to detect the hole defects, and the target goes through Sobel processing to make the blemish outline more obvious. The inner circle and open circle 1 detection processes are used to detect the dirty defects, while open circle 2 is used to detect burr defects. Figure 15 is a flowchart for the full detection process and the steps associated with the images’ processing. First, the inner-circle region is sliced out through the image segmentation process, and the binarization process is used to treat the segmented region. When the gray value of the treated area is smaller than the critical value, the area is recognized as a hole and is shown as black. On the other hand, the gray value of the treated area was larger than the critical value, it would be seen as background and shown as white. Afterwards, the technology of connected components is used for further processing, which distinguishes the adjacent pixels in the same block. When the calculated pixels were larger than 200, the treated area was recognized as the defect. For the inner circle, hollow circle 1, and hollow circle 2 detection processes, the images are processed by segmentation and binarization to proceed with the detection. When the gray value of a detection area is smaller than the critical value, it is recognized as background and shown as black. On the other hand, if the gray value of a detection area is greater than the critical value, it is recognized as dirt, burrs, or uneven thickness and shown as white. Furthermore, the technology of connected components is used to divide the adjacent pixels into the same block. As long as the region of defect pixels is larger than 200, the treated area is displayed as a defect, and the processing time of the internal detection process of a paper cup is 116 ms.

**Figure 16:**
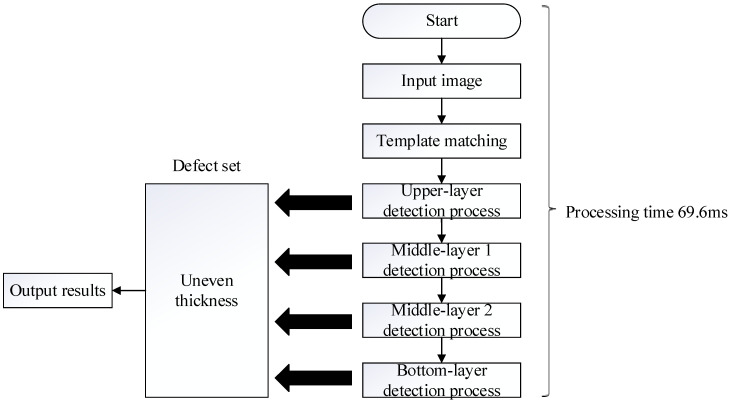
External detection processes of a paper cup.

**Figure 17:**
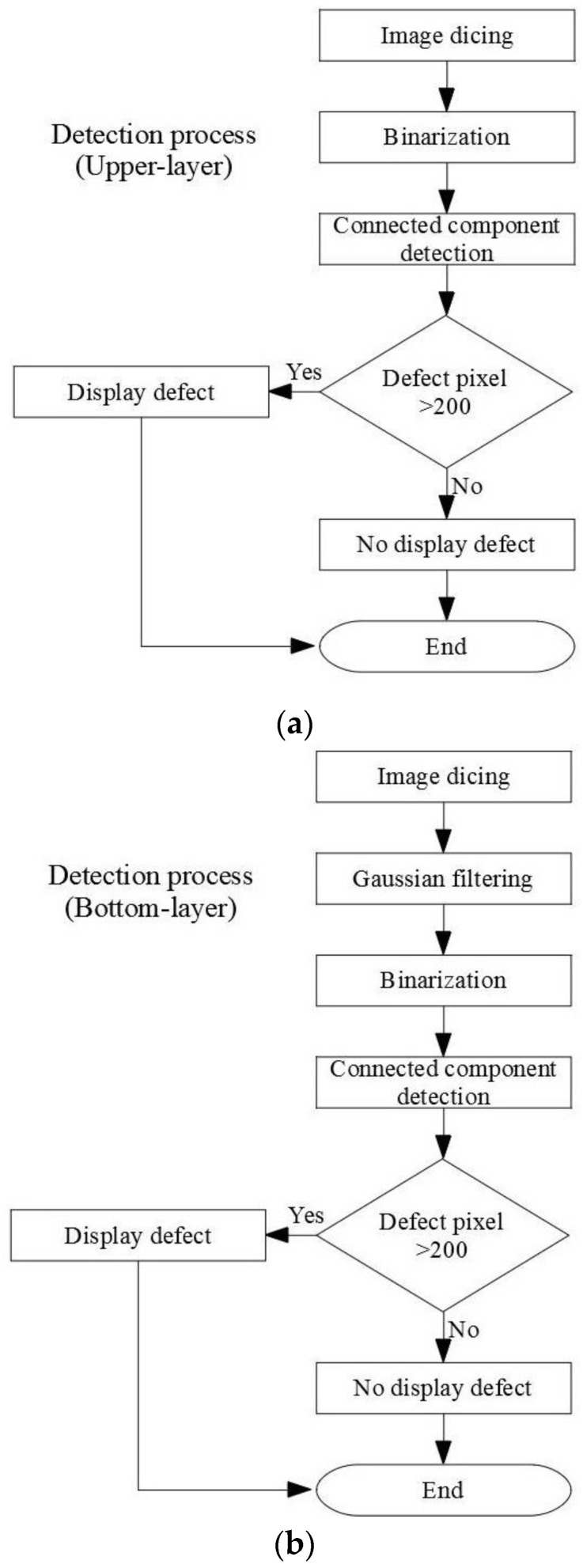

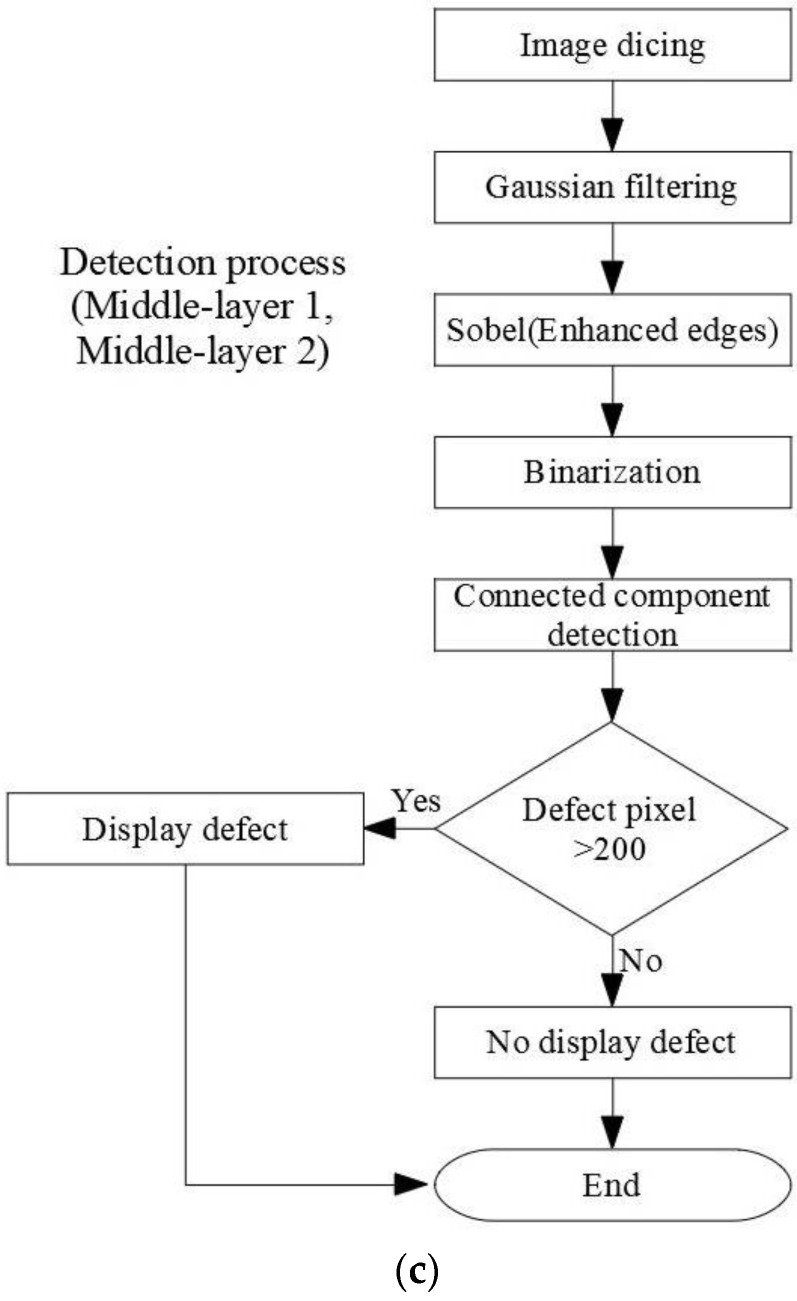
Detection processes of (**a**) upper on the outside, (**b**) bottom on the outside, and (**c**) middle 1 and middle 2 on the outside of a paper cup.

**Figure 18:**
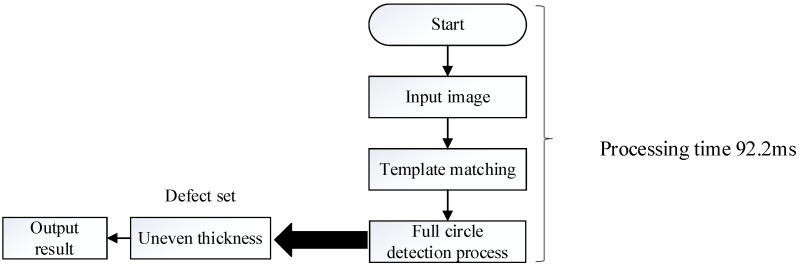
Bottom detection process of a paper cup.

**Figure 19:**
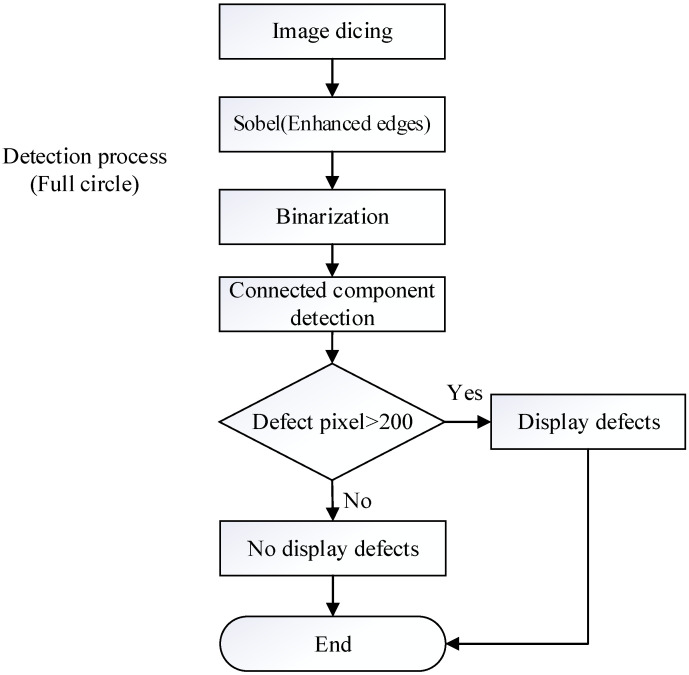
Full circle for the bottom detection process of a paper cup.

Figure 16 shows the external detection processes of the manufactured paper cups; the captured image template was matched with the target, and the search paper cup target was mainly locked. The first detection process is carried out to detect the uneven thickness defects, and next, the bottom inspection process is then carried out to detect the uneven thickness defects. For the two detection processes, the upper detection process, the middle 1 detection process, the middle 2 detection process, and the final bottom detection process are carried out, all of which are used to detect the uneven thickness defects. As the upper detection process shows in Figure 17, Figure 18 and Figure 19, the upper range of the segmented image is processed using Gaussian filtering, and then the bright, uneven surface is evenly diffused. Furthermore, the Sobel processing is used to go through and highlight the contours of the defects, and finally, the binarization process is applied to the image. After binarization, when the gray value of an area is smaller than the defined critical value, black is displayed, and black is the background. When the gray value is larger than the defined critical value, white is displayed, and white is the defect of the uneven thickness. After performing these processes on the target, the uneven thickness defects can really be separated from the background. 

Furthermore, the connected component is used to divide the adjacent pixels into the same block. When the defect pixels in an area are larger than 200, the detection result for this area is displayed as the defect. In the detection processes of middle-layer 1 and middle-layer 2, the images are segmented into blocks, and the uneven surface and the bright uneven surface become smoother and spread evenly through Gaussian filtering. The Sobel process is then used to make the blemish outlines of the defects more obvious, and finally, the binarization process is processed on these images. When the gray value is less than the critical values of middle-layer 1 and middle-layer 2, it displays black and is recognized as the background. When the gray value is larger than the critical values of middle 1 and middle 2, it displays white, and white is of uneven thickness. Then the connected component process is used to divide the adjacent pixels into the same block. When it is judged that the defect pixels in an area are larger than 200, the defect result is displayed.

The bottom detection process mainly uses Gaussian filtering so that the bright, uneven surface becomes evenly diffused. The area where the gray value is smaller than the critical value is recognized as background and displays black. On the other hand, the area where the gray value is larger than the critical value is recognized as having uneven thickness and shows as white. Therefore, white is the region of the uneven thickness, and the region of defect is needed to be separated from the background. Furthermore, the connected component process is used to divide the adjacent pixels into the same block. When the region of defect pixels is larger than 200, the region is recognized as a defect and is displayed. The processing time of the outside detection process of the paper cup is only 69.6 ms. 

As shown in Figure 18, this step is to perform template matching on the captured image, which is used to locate the paper cup target and perform full-circle detection. This detection process is mainly performed to detect the uneven thickness. As shown in Figure 19, after the image is divided into a full circle, the Sobel process is used to make the edges of the defects more prominent, and the binarization process is performed on the image. When the gray value of an area is less than the critical value, it displays black and is recognized as the background. On the other hand, when the gray value of an area is larger than the critical value, it displays white, and this area is recognized as having an uneven thickness. Furthermore, the connected component process is used to segment the adjacent pixels into the same block. When the defect pixels in an area are judged to be larger than 200, the defect of the uneven thickness is displayed. The processing time for the bottom detection of the paper cup is 92.2 ms.

### 3.4. Detection Results of Defects in Paper Cups

Since the captured images of all the detected cups performed the internal, external, and bottom detection processes, the processing and recognition times of each image captured from the cups are different. Therefore, we use the processing time of 20 samples to calculate the average processing time. As Table 4 shows, the time to detect the internal defect was ranged from 102 to 151 ms, the time to detect the external defect was ranged from 52 to 74 ms, and the time to detect the bottom defect was ranged from 88 to 99 ms, respectively. Therefore, the average internal, external, and bottom defect detection times were 116, 69.6, and 92.2 ms, as shown in Table 4. In this research, the detection rates of the investigated system for internal, external, and bottom defect detection processes are 100%, and the average processing and calculation time of the defect detection for the paper cups is 277.8 ms. These results also prove that the combination of the Gaussian filter, Sobel operator, Binarization process, and connected components can enhance the recognition effects of the images captured by our self-assembled detection system.

In the past, Park et al. proposed a detection method by using a deep learning classifier to detect the four kinds of defects in the paper cups, and they used a Charge Coupled Device camera and diffused LED lights to capture the images of the produced paper cups, but their detection rate was only 96.5% [26]. Bing used the technology of machine vision to detect the defects on the paper cups, and the detection rates were in the range of 96–100% [27]. In this study, the captured images and the detection images for different positions and different defects are compared and shown to prove the defection effects of the investigated optical automatic detection system combined with the Gaussian filter, Sobel operator, Binarization process, and connected component. Finally, the investigated system was actually used to process the internal capturing, external capturing, and bottom capturing for tens of thousands of paper cups on the production line. We found that the investigated detection technology had a detection accuracy rate of 99.7% for the different defects. This result proves that the investigated system has a high detective efficiency on the defects of the manufactured paper cups. To further prove the detective efficiency of the investigated system, the images for different defects of the manufactured paper cups are shown below.

**Figure 20:**
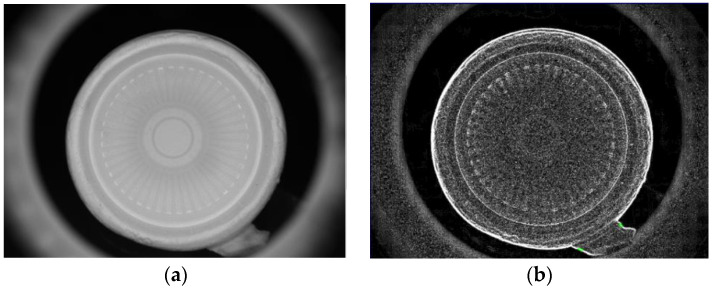
(**a**) Original image and (**b**) the processing image of internal burrs in a paper cup, green color is used to mark the defect region.

**Figure 21:**
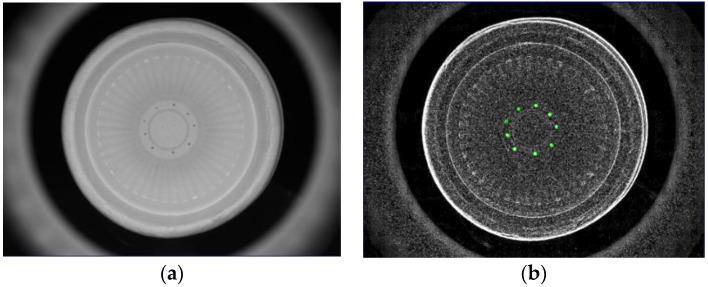
(**a**) Original image and (**b**) the processing image of the internal dirt in a paper cup, green color is used to mark the defect region.

**Figure 22:**
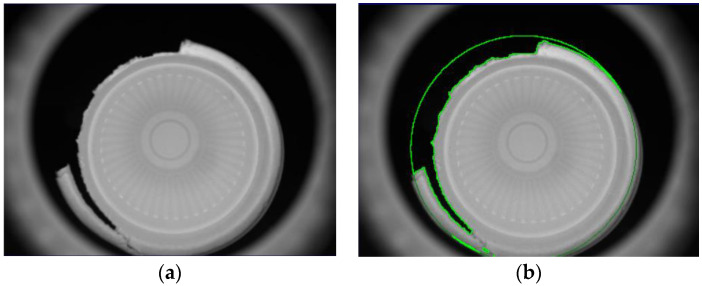
(**a**) Original image and (**b**) the processing image of the internal hole in a paper cup, green color is used to mark the defect region.

**Figure 23:**
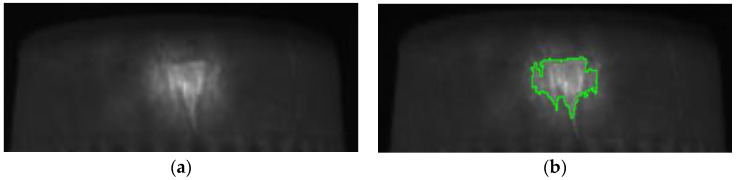
(**a**) Original image and (**b**) the processing image of the external upper layer uneven thickness of a paper cup, green color is used to mark the defect region.

**Figure 24:**
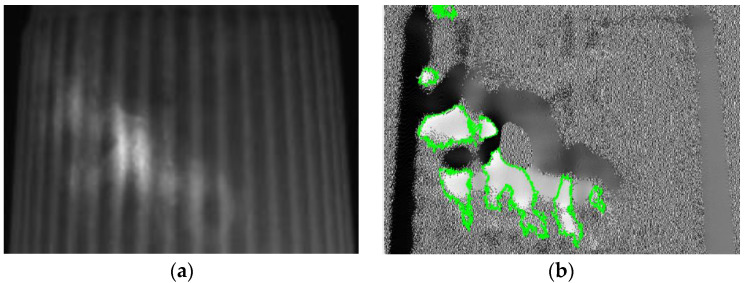
(**a**) Original image and (**b**) the processing image of the external middle-layer 1 uneven thickness of a paper cup, green color is used to mark the defect region.

**Figure 25:**
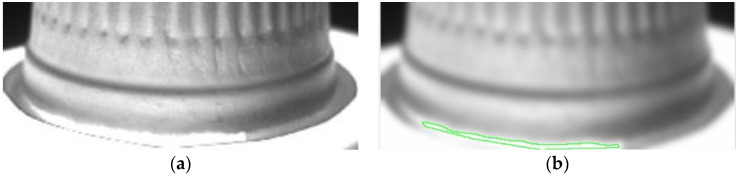
(**a**) Original image and (**b**) the processing image of the external bottom uneven thickness of a paper cup, green color is used to mark the defect region.

**Figure 26:**
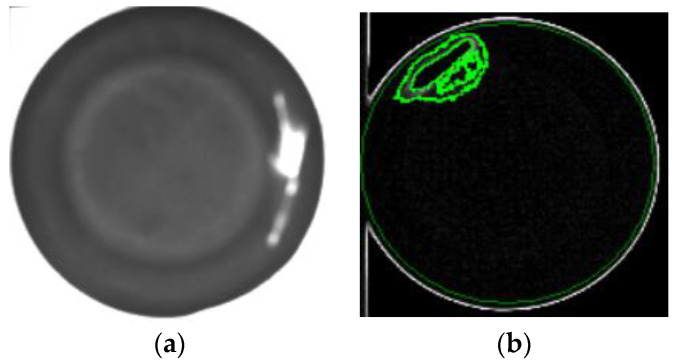
(**a**) Original image and (**b**) the processing image of the bottom uneven thickness of a paper cup, green color is used to mark the defect region.

In order to prove that the investigated self-assembly imaging capture system, combined with the image processing software of the Gaussian filter, Sobel operator, Binarization process, and connected component, can have a high detection rate, the produced paper cups with different defects at different positions are presented below. Figure 20a shows the original image of a paper cup; apparently, the internal burrs really exist in this cup. Figure 20b shows that the internal burr in the cup was really detected, and the edge of the burr was really defined in the processing image, which proves that the investigated technology can really detect the internal burr in a cup. Figure 21a is the original image of a paper cup, and the dirt really existed in the interior of this detected paper cup, and Figure 21b shows that the internal dirt in the cup was really detected and defined in the processing image. Figure 22a shows the captured image of a cup with a hole, and Figure 22b also shows that the internal hole was really detected and the edge of the hole was really defined in the processing image. Figure 23a,b are the original image and the processing image of uneven thickness of the external upper layer of a paper cup, and Figure 24a,b are the original image and the processing image of uneven thickness of the middle layer of a paper cup. The Figure 24a,b show that the uneven thickness of a paper cup at different positions was really detected and defined, and the edge of the uneven thickness was really defined in the processing image. Furthermore, Figure 25a,b are the original image and the processing image of the external upper layer uneven thickness of a paper cup, and Figure 26a,b are the original image and the detection image of the bottom uneven thickness of a paper cup. The uneven thickness of a paper cup at different positions could also be defined. These results prove that our investigated system is practical for detecting the defects in the paper products.

## 4. Conclusions

In this research, an automatic optical detection system was designed to successfully detect the defects of the manufactured paper cups, including dirt, burrs, holes, and uneven thickness. The visual detection takes a long average of 2.5 s, but the average internal, external, and bottom defect detection times were 116, 69.6, and 92.2 ms. The average processing and calculation time for the detection of defects in the paper cups was 277.8 ms, which was nine times faster than the use of machine vision detection. The investigated system was actually used to detect tens of thousands of paper cups on the production line, with a detection accuracy rate of 99.7%. In conclusion, we argue that machine vision detection provides a powerful way to increase defect detection efficiency and resolve the visual fatigue problem of manual detection. The detection results showed that when the defective pixels are used to convert the defective areas, they can meet the actual areas of the defective paper products according to the requirements of human customization.

## Figures and Tables

**Table 1:** Calculation results for the measured defection area of 25 mm^2^.

WD (mm)	Pixel Area (Pixels)	Identified Defect Area (mm^2^)	Actual Defect Area (mm^2^)	Error (%)
310	2959	22.5	25	10
320	2803	22.7	25	9.2
330	2620	22.6	25	9.7
340	2472	22.6	25	9.6
350	2302	22.3	25	10.8
360	2218	22.7	25	9.0
370	2087	22.6	25	9.6
380	1977	22.6	25	9.7
390	1878	22.6	25	9.6
400	1764	22.3	25	10.7
Average error 9.8				

**Table 2:** Calculation results for the measured defection area of 100 mm^2^.

WD (mm)	Pixel Area (Pixels)	Identified Defect Area (mm^2^)	Actual Defect Area (mm^2^)	Error (%)
310	11,972	91	100	9.0
320	11,322	91.7	100	8.3
330	10,571	91.1	100	8.9
340	10,069	92.1	100	7.9
350	9435	91.4	100	8.6
360	9037	92.6	100	7.4
370	8508	92.1	100	7.9
380	8060	92.1	100	7.9
390	7675	92.3	100	7.7
400	7256	91.8	100	8.2
Average error 8.2				

**Table 3:** Calculation results for the measured defection area of 400 mm^2^.

WD(mm)	Pixel Area (Pixels)	Identified Defect Area (mm^2^)	Actual Defect Area (mm^2^)	Error (%)
310	48,034	365.1	400	8.7
320	45,295	366.9	400	8.3
330	42,427.3	365.5	400	8.6
340	40,361.6	369.1	400	7.7
350	37,906.8	367.3	400	8.2
360	36,136.6	370.5	400	7.4
370	34,123.4	369.5	400	7.6
380	32,402.2	370.1	400	7.5
390	30,946.8	372.3	400	6.9
400	29,343.4	371.4	400	7.2
Average error 7.8				

**Table 4:** Results of the operation time for the defect detections of paper cups.

Sample	Internal (ms)	External (ms)	Bottom (ms)
1	105	72	95
2	112	74	94
3	102	70	93
4	102	69	92
5	106	74	91
6	144	69	90
7	114	71	91
8	130	70	90
9	106	76	92
10	110	66	89
11	110	52	91
12	108	71	92
13	114	71	93
14	104	71	95
15	101	70	93
16	151	69	90
17	105	68	94
18	105	69	99
19	144	73	92
20	147	66	88
Average	116	69.6	92.2
Total		277.8	

## Data Availability

Not applicable.
